# Ulnar nerve stability-based surgery for cubital tunnel syndrome via a small incision: a comparison with classic anterior nerve transposition

**DOI:** 10.1186/s13018-015-0267-8

**Published:** 2015-08-06

**Authors:** Ho-Jung Kang, Il-Hyun Koh, Yong-Min Chun, Won-Taek Oh, Kwang-Ho Chung, Yun-Rak Choi

**Affiliations:** Department of Orthopedic Surgery, Severance Hospital, Yonsei University College of Medicine, Yonseiro 50-1, Seodaemun-gu, Seoul 120-752 Republic of Korea

## Abstract

**Objective:**

The purpose of this study was to compare the clinical outcomes of ulnar nerve stability-based surgery via a small incision with those of classic anterior transposition of the ulnar nerve for cubital tunnel syndrome.

**Methods:**

From March 2008 to December 2013, 107 patients with cubital tunnel syndrome underwent simple decompression or anterior transposition via a small incision, according to an ulnar nerve stability-based decision based on an assessment of intraoperative ulnar nerve stability (group A, *n* = 51), or anterior transposition via a classic incision (group B, *n* = 56). Clinical outcome was assessed using grip and pinch strength, two-point discrimination, the mean of the disabilities of arm, shoulder, and hand (DASH) survey, and the modified Bishop scale.

**Results:**

At the final follow-up, all outcome measures improved significantly in both groups and there were no significant differences between the two groups. However, there were fewer operation-related complications in group A (one revision surgery) than in group B (one superficial infection, two painful scars, and five cases of numbness at the medial elbow).

**Conclusions:**

Outcomes after the ulnar nerve stability-based approach and anterior transposition were similar, although more patients experienced operation-related complications after anterior transposition via a classic incision. Making an ulnar nerve stability-based decision to perform either simple decompression or anterior transposition via a small incision seems to be a better strategy for patients with cubital tunnel syndrome.

## Background

Cubital tunnel syndrome is the second most common upper-extremity compressive neuropathy, after carpal tunnel syndrome. Operative treatment is indicated after failure of conservative management. Studies have investigated the outcomes of operative techniques for treating cubital tunnel syndrome including open [1, 2] or endoscopic [3, 4] simple decompression; subcutaneous [5–7], intramuscular [8], or submuscular [2, 9] anterior transposition; and medial epicondylectomy [6, 10, 11]. However, there is no consensus regarding the best surgical technique for the condition.

The most commonly used procedure for operative treatment of cubital tunnel syndrome is anterior transposition of the ulnar nerve, as this procedure can decrease the tension of the ulnar nerve that occurs with elbow flexion [12–14]. However, extensive dissection is needed to transpose the ulnar nerve, which may compromise its vascularity [15]. In contrast, simple decompression only relieves direct compression of the nerve and does not require extensive dissection; however, it cannot be used to treat dynamic ulnar nerve compression [14]. Another concern of simple decompression is that transposition after a failed simple decompression is likely to be more difficult.

A recent meta-analysis of four randomized trials showed no differences in motor nerve conduction velocities or clinical outcome scores for patients with cubital tunnel syndrome treated with either simple decompression or ulnar nerve transposition [15]. However, two out of the four randomized studies specifically excluded patients with nerve subluxation [16, 17], limiting the applicability of these results to patients with nerve instability. Apart from the results of the above meta-analysis, Bimmer and Meyer reported improved outcomes in patients with ulnar nerve instability following anterior transposition [18]. The need for a tailored surgical approach for each patient is on the rise, and a retrospective study by Keith et al. found excellent clinical outcomes for a tailored approach of simple decompression or anterior subcutaneous transposition based on intraoperative ulnar nerve stability assessment [19]. However, data comparing that approach to classic anterior transposition of the nerve is lacking.

The purpose of this study was to investigate whether the subjective and objective outcomes after making an ulnar nerve stability-based surgical decision to perform either simple decompression or anterior ulnar nerve transposition depending on intraoperative ulnar nerve stability via a small incision were comparable to those of classic anterior transposition of the ulnar nerve.

## Materials and methods

From March 2008 to December 2013, 163 patients with clinically and electrodiagnostically confirmed cubital tunnel syndrome underwent operative treatment for cubital tunnel syndrome. Electrodiagnostic studies were conducted and interpreted by a professional rehabilitation doctor at our institution. We recommended operative treatment if patients presented with intrinsic atrophy or significant hand weakness and had clinical symptoms of tingling, pain, or weakness after at least 2 months of conservative treatment, such as night splinting and tendon gliding exercises.

The inclusion criteria comprised surgically treated cubital tunnel syndrome and follow-up data that were available for a minimum of 1 year after surgery. The exclusion criteria were as follows: electrodiagnostically silent cubital tunnel syndrome, cubitus valgus, osseous canal deformity from previous trauma or osteophytes of the elbow joint, previous surgery for cubital tunnel syndrome, associated cervical radiculopathy, carpal tunnel syndrome, ulnar tunnel syndrome, thoracic outlet syndrome, diabetes mellitus, hypothyroidism, worker’s compensation issues, and follow-up data unavailability for a minimum of 1 year after surgery.

**Fig. 1:**
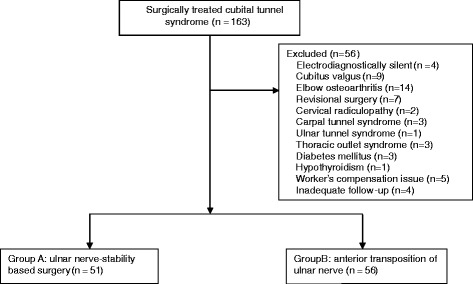
The CONSORT diagram of enrollment and analysis in this study

Based on these criteria, four patients with electrodiagnostically silent cubital tunnel syndrome, nine patients with cubitus valgus, 14 patients with elbow osteoarthritis, seven patients requiring revision surgery, 13 patients with one of the associated diseases mentioned above, and five worker’s compensation patients were excluded. Four patients were lost to follow-up. Consequently, 56 patients were excluded, and 107 patients were available for the study (Fig. 1). Among our study population, 12 patients had bilateral cubital tunnel syndrome. In these patients, we analyzed only the dominant extremity. We then had 51 patients who underwent an ulnar nerve stability-based approach involving either simple decompression (*n* = 37) or anterior transposition (*n* = 14) via a small incision (group A) and 56 patients who underwent anterior subcutaneous transposition of the ulnar nerve via a classic incision (group B). There was a distinct time period for each type of operation. Briefly, we performed anterior transposition of the ulnar nerve via a classic incision earlier in the duration of the study and changed the technique to an ulnar nerve stability-based approach via a small incision in June 2010. Group A included 32 men and 19 women with a mean age of 38.3 ± 15.0 years (range, 20–68 years) at the time of surgery. The duration of symptoms to surgery was 24.1 ± 31.2 months (range, 3–120 months). The mean follow-up period after the operation was 30.2 ± 10.8 months (range, 12–48 months). Group B included 37 men and 19 women with a mean age of 35.7 ± 16.7 years (range, 19–66 years) at the time of surgery. The duration of symptoms to surgery was 23.0 ± 26.8 months (range, 5–96 months). The mean follow-up period after the operation was 34.1 ± 13.2 months (range, 12–60 months; Table 1). Our institutional review board approved the study and waived the requirement for informed consent.

**Table 1:** Baseline demographic and clinical characteristics

Characteristic	Group A (*n* = 51)	Group B (*n* = 56)	*p*
Mean age (years)	38.3 ± 15.0	35.7 ± 16.7	0.408
Male gender, *n* (%)	32 (63)	37 (66)	0.720
Mean duration of symptoms (months)	24.1 ± 31.2	23.0 ± 26.8	0.845
Preoperative stability of ulnar nerve, *n* (%)			0.650
Stable	41 (80)	43 (77)	
Unstable	10 (20)	13 (23)	
Dellon grade, *n* (%)			0.796
I	9 (18)	11 (20)	
II	27 (53)	26 (46)	
III	15 (29)	19 (34)	
MCV at elbow segment (m/s)	39.5 ± 8.4	38.4 ± 8.6	0.50
Surgical procedure, *n* (%)			<0.001
Simple decompression	37 (73)	0 (0)	
Anterior transposition	14 (27)	56 (100)	
Mean follow-up after operation (months)	30.2 ± 10.8	34.1 ± 13.2	0.105

*MCV* motor nerve conduction velocity at the elbow segment

Dellon staging was applied in order to grade the preoperative severity of ulnar neuropathy [20]. According to this staging system, patients with intermittent paresthesia and subjective weakness are classified as having mild ulnar nerve compression (grade I). Patients who have moderate compression show intermittent paresthesia and measurable weakness in pinch and grip strength (grade II). Patients with persistent paresthesia, abnormal two-point discrimination, and measurable weakness in pinch and grip strength with intrinsic atrophy are classified as having severe compression (grade III). Accordingly, nine patients were rated as grade I, 27 as grade II, and the remaining 15 as grade III in group A; similarly, 11 patients were rated as grade I, 26 as grade II, and the remaining 19 as grade III in group B (Table 1).

An independent observer (BRK) blinded to the method of operation performed the preoperative and postoperative assessments. Each patient was assessed for grip and pinch strength and two-point discrimination (2PD) and completed the disabilities of arm, shoulder, and hand (DASH) survey preoperatively and at each follow-up [21]. Pinch and grip strength were measured using baseline hydraulic pinch and grip dynamometers. The clinical outcome at the final follow-up was based on the Bishop rating system, which assesses subjective and objective parameters [9]. Subjective parameters included severity of residual symptoms (asymptomatic, 3; mild, 2; moderate, 1; severe, 0), subjective improvement compared with the preoperative period (better, 2; unchanged, 1; worse, 0), and preoperative and postoperative work status (working previous job, 2; changed job, 1; not working, 0). Objective parameters were grip strength compared with the normal side (80 % or more, 1; less than 80 %, 0) and sensory measurement of static two-point discrimination (6 mm or less, 1; more than 6 mm, 0). The score was defined as excellent (8 to 9), good (5 to 7), fair (3 to 4), and poor (0 to 2).

### Surgical technique

Under general anesthesia, the patient was placed in a supine position with the affected arm supported by a hand table and sterilely prepped and draped. After exsanguination of the limb with a sterile tourniquet, the shoulder was placed at 90° of abduction and slight external rotation and the medial epicondyle and olecranon were marked.

**Fig. 2:**
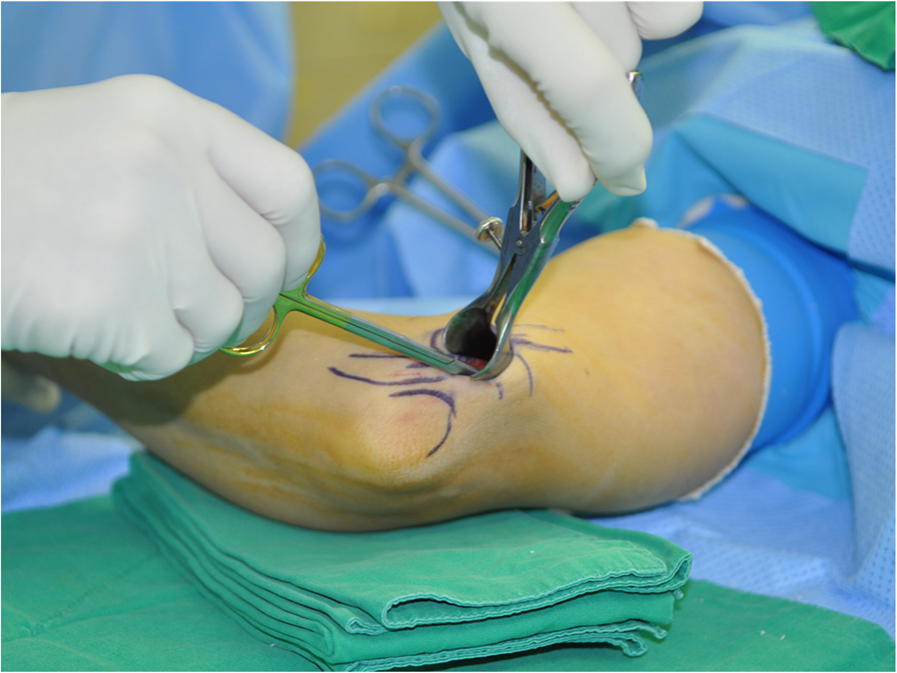
While introducing and opening a long nasal speculum over the brachial fascia, the proximal nerve compression structures including the arcade of Struthers were completely released

**Fig. 3:**
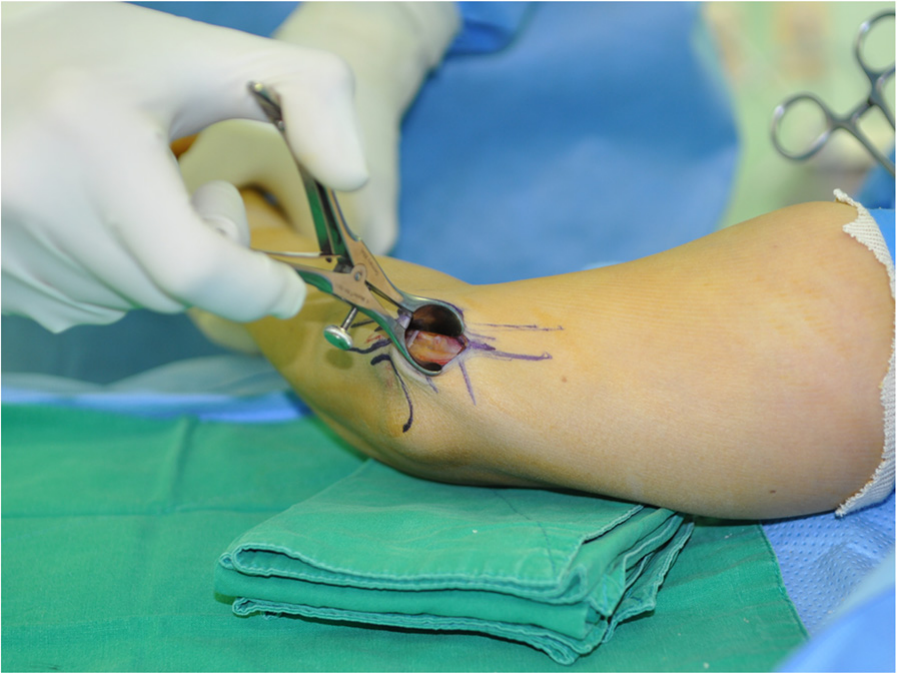
After releasing the proximal nerve compression structures, Osborne’s ligament, Osborne’s fascia, and the deep flexor-pronator aponeurosis were sequentially released

**Fig. 4:**
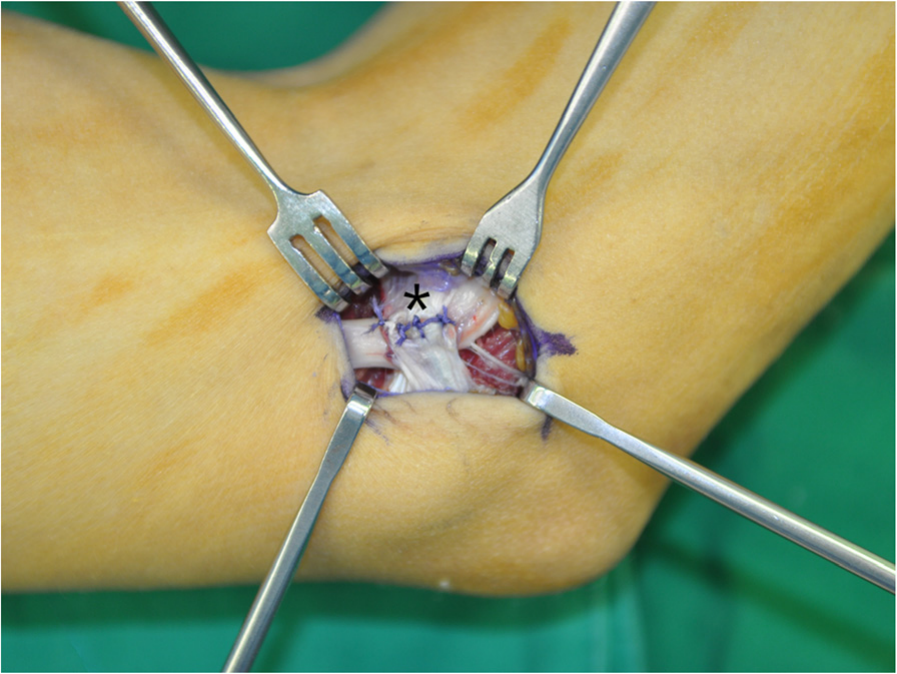
In patients with an unstable ulnar nerve, the nerve was anteriorly transposed, and a fascial sling (*) was created

In group A, a 2.5-cm longitudinal skin incision was made between the medial epicondyle and the olecranon. Then, the subcutaneous tissues were gently and carefully separated with dissecting scissors. With the help of mini retractors, the ulnar nerve was located by releasing the brachial fascia just proximal to the cubital tunnel. Blunt dissection was carried out proximally using a curved mosquito hemostat to create a cavity between the subcutaneous tissue and the brachial fascia. A Cobb elevator was then gently introduced into this cavity to extend it at least 8 cm proximal to the medial epicondyle. A long nasal speculum was introduced into the cavity, and the brachial fascia and arcade of Struthers were released under direct visualization (Fig. 2). To allow the operating light to reach the deep operating field, the shoulder of the patient was adducted about 20°, and the beam of the light was almost parallel to the upper arm. After removing the nasal speculum, Osborne’s ligament was released. Then, a distal cavity was created between the subcutaneous tissue and Osborne’s fascia, followed by the release of Osborne’s fascia and the deep flexor-pronator aponeurosis. A short nasal speculum was introduced at that moment to assist with clear visualization of the structures (Fig. 3). Only the superficial surface of the nerve was exposed, and neurolysis was not performed to decrease the possibility of nerve subluxation. After complete release of all potential sources of structural nerve compression, the stability of the ulnar nerve was tested by moving the elbow through the full range of motion. If the nerve remained within the cubital tunnel throughout elbow flexion, it was considered stable. If the nerve displaced onto the medial epicondyle during flexion or if it did not sit well within the cubital tunnel, it was considered unstable. In such cases where instability was identified intraoperatively, the skin incision was extended 1 cm proximally and distally to transpose the nerve anteriorly. The soft tissue above the flexor-pronator muscle group was elevated, and the ulnar nerve was then carefully lifted from its bed with its accompanying longitudinal vascular supply intact. Segmental feeding vessels were identified and ligated to prevent tethering. Neurolysis of the posterior motor branches from the main ulnar nerve was performed to allow adequate anterior transposition if there was tension. The medial intermuscular septum was also excised as part of the anterior transposition. A fascial sling raised from the underlying muscle fascia was created to prevent slippage of the nerve after transposition (Fig. 4).

In group B, a 10-cm incision was placed behind the medial epicondyle. All points of ulnar nerve compression were completely released as described earlier, and care was taken to protect branches of the medial antebrachial cutaneous nerve. The soft tissue above the flexor-pronator muscle origin was elevated, and the ulnar nerve was then lifted from its bed as described earlier. The medial intermuscular septum was also excised, and a fascial sling raised from the underlying muscle fascia was created.

After skin closure, a soft dressing and an elastic bandage were applied. Early movement of the fingers, wrist, forearm, elbow, and shoulder was encouraged.

### Statistical analysis

SPSS Statistics version 18.0 (SPSS, Inc, IBM®, Chicago, IL, USA) was used for statistical analyses. Group results were compared using Pearson’s chi-squared test or Fisher’s exact test for categorical variables and Student’s *t* test for continuous variables. The level of significance was set at *p* < 0.05.

## IRB approval

This study was approved by Severance Hospital Institutional Review Board: 4-2012-0227.

## Results

Baseline patient characteristics, duration of symptoms, Dellon’s grade, initial grip strength, initial pinch strength, initial 2PD, and initial DASH scores did not differ significantly between groups (Table 1).

In group A, grip strength increased from a mean of 17.9 ± 8.6 to 31.9 ± 10.9 kg (*p <* 0.001), and pinch strength increased from a mean of 3.3 ± 1.8 to 4.1 ± 2.0 kg (*p = 0*.008) at the final follow-up. The mean two-point discrimination improved from 6.4 ± 2.3 to 3.2 ± 1.1 mm (*p <* 0.001). The mean DASH score improved from 35.1 ± 21.3 to 11.0 ± 10.0 (*p* < 0.001; Table 2). According to the modified Bishop rating system, excellent results were observed in 29 patients, good in 19, and fair in three. One patient with a fair result, who had undergone in situ decompression as an index procedure, underwent anterior subcutaneous transposition of the nerve at 15 months postoperatively and reported improved symptoms after revision surgery. Based on the modified Bishop rating system, this patient’s score was 5 at the final follow-up. There were no other complications related to the operation in group A.

**Table 2:** Clinical outcomes at last follow-up

	Group A	Group B	*p* value
Grip strength (kg)			<.001
Preoperative	17.9 ± 8.6	20.8 ± 8.8	0.080
Last FU	31.9 ± 10.9	31.8 ± 11.1	0.931
Pinch strength (kg)			
Preoperative	3.3 ± 1.8	3.2 ± 1.6	0.642
Last FU	4.1 ± 2.0	4.0 ± 2.0	0.777
2PD (mm)			
Preoperative	6.4 ± 2.3	6.2 ± 2.7	0.566
Last FU	3.2 ± 1.1	3.1 ± 1.1	0.560
DASH score			
Preoperative	35.1 ± 21.3	31.4 ± 18.1	0.327
Last FU	11.0 ± 10.0	10.8 ± 8.3	0.919
Final outcome, *n* (%)			0.580
Excellent	29 (57)	35 (63)	
Good	19 (37)	16 (29)	
Fair	3 (6)	5 (8)	

*2PD* two-point discrimination, *DASH* disability of arm, shoulder, and hand, *PO* postoperation

In group B, grip strength increased from a mean of 20.8 ± 8.8 to 31.8 ± 11.1 kg (*p* < 0.001) and pinch strength increased from a mean of 3.2 ± 1.6 to 4.0 ± 2.0 kg (*p* = 0.003) at the final follow-up. The mean two-point discrimination improved from 6.2 ± 2.7 to 3.1 ± 1.1 mm (*p* < 0.001). The mean DASH score improved from 31 ± 18 to 11 ± 8 (*p* < 0.001; Table 2).

According to the modified Bishop scale, excellent results were observed in 35 patients, good in 16, and fair in five. There were eight complications: superficial infection in one, painful scarring in two, and numbness at the medial elbow in five (Table 3).

**Table 3:** Operation-related complications

	Group A	Group B	*p* value
Superficial infection	0	1	
Revision surgery	1	0	
Painful scar	0	2	
Numbness at medial elbow	0	5	
Total number of complications	1	8	0.033

There were no statistically significant differences between the outcomes of the two groups at the final follow-up. However, the rate of complications was higher in group B than in group A (*p* = 0.033).

## Discussion

Although various surgical techniques have been introduced to treat cubital tunnel syndrome, the optimal surgical treatment is unclear. Anterior transposition of the ulnar nerve is the most commonly performed procedure for operative treatment of cubital tunnel syndrome, as it can address traction on the ulnar nerve during flexion of the elbow [12, 14]. To transpose the ulnar nerve, extensive dissection is necessary, which puts the vascularity of the nerve at risk and increases the risk of injury to the medial antebrachial cutaneous nerve. In contrast, simple decompression is considered to be an effective method for the treatment of cubital tunnel syndrome with advantages over anterior transposition, including cost-effectiveness, preservation of the blood supply to the nerve, and shorter operative and rehabilitation times [1, 15, 22, 23]. When surgeons choose simple decompression or anterior transposition, the presence of ulnar nerve subluxation with elbow flexion is one of the factors that influence the choice [18, 24]. Although making the ulnar nerve stability-based surgical decision to perform simple decompression or anterior transposition for patients with cubital tunnel syndrome showed favorable clinical outcomes [19], there have been no investigations comparing this surgical approach to classic anterior transposition of the ulnar nerve. In this study, we asked whether the subjective and objective outcomes after an ulnar nerve stability-based surgical approach via a small incision were comparable to those of anterior transposition of the ulnar nerve via a classic incision.

In this study, functional restoration after the ulnar nerve stability-based approach was similar to that after classic anterior transposition of the ulnar nerve, as measured by outcome instruments including grip strength, two-point discrimination, DASH score, and modified Bishop score. Similar to a study conducted by Keith and Wollstein [19], use of the ulnar nerve stability-based approach resulted in 94 % of patients (48 of 51) experiencing improvement in upper limb function based on the modified Bishop score. Previously, no statistically significant differences in clinical outcomes were observed between simple decompression and anterior transposition in patients without ulnar nerve instability [15–17]. This indicates that anterior transposition of the ulnar nerve seems to be necessary only in patients with ulnar nerve instability, so as to eliminate the symptoms of a painful subluxation of the nerve.

One of the most common complications following cubital tunnel surgery is injury to the posterior branch of the medial antebrachial cutaneous nerve, which is encountered in all surgical approaches to the ulnar nerve. Injury to the nerve could cause painful neuroma, hyperesthesia, hyperalgesia around the medial elbow, and painful scarring [25, 26]. In this study, 16 % of patients (7 of 56) who underwent anterior transposition of the ulnar nerve via a classic incision experienced painful scarring (two patients) and numbness at the medial elbow (five patients); these complications were likely due to medial antebrachial cutaneous nerve injury during the surgical procedure. However, there were no injuries to the medial antebrachial cutaneous nerve in patients who underwent the ulnar nerve stability-based approach via a small incision. One possible explanation is that the small incision was made proximal to the medial epicondyle and the medial antebrachial cutaneous nerve was elevated without exposure within the elevated skin flap. In contrast, the classic technique for anterior transposition of the ulnar nerve requires a greater amount of tissue dissection than the mini-open technique, which increases the risk of injury to the medial antebrachial nerve. Concerns have been raised regarding the likelihood of ulnar nerve subluxation after simple decompression; however, there were no patients in this study who complained of symptomatic subluxation after simple decompression. This may have been due to there being only superficial release of the ulnar nerve, not neurolysis of the nerve as suggested previously [27]. In this study, one patient who underwent simple decompression and had a fair result was subject to a secondary surgery, anterior transposition of the nerve, at 15 months postoperatively. The secondary surgery may have been more difficult due to the presence of scar tissues; nevertheless, it seems that it would have been much easier after simple decompression than after anterior transposition, as simple decompression requires minimal dissection.

There are several limitations to this study. First, we used two outcome measures, the modified Bishop scale and the DASH score, to estimate the clinical outcomes after surgical treatment; however, there is no reliable, reproducible, and valid outcome measure for cubital tunnel syndrome [24]. An instrument reflecting patient-reported outcomes and satisfaction in combination with quantitative clinical findings is needed for standardized assessment of patients with cubital tunnel syndrome to accurately assess the clinical outcomes of surgical treatment for this common ulnar neuropathy. Second, this study includes only a limited number of cases with a relatively short-term follow-up period. Third, in our series, the interval between the onset of symptoms and surgeries was relatively longer than other studies, likely because our institution is a tertiary referral hospital and our patients were requested to recall the first time they experienced nerve symptoms even if they had a symptom-free period between each episode of symptoms.

Based on the results of our study, we presume that ulnar nerve stability-based surgery for idiopathic cubital tunnel syndrome involving either simple decompression or anterior transposition via a small incision is a safe and effective strategy for surgical treatment following the failure of non-operative management.
